# Deep ensemble learning-driven fully automated multi-structure segmentation for precision craniomaxillofacial surgery

**DOI:** 10.3389/fbioe.2025.1580502

**Published:** 2025-05-08

**Authors:** Jiahao Bao, Zongcai Tan, Yifeng Sun, Xinyu Xu, Huazhen Liu, Weiyi Cui, Yang Yang, Mengjia Cheng, Yiming Wang, Congshuang Ku, Yuen Ka Ho, Jiayi Zhu, Linfeng Fan, Dahong Qian, Shunyao Shen, Yaofeng Wen, Hongbo Yu

**Affiliations:** ^1^ Department of Oral and Craniomaxillofacial Surgery, Shanghai Ninth People’s Hospital, Shanghai Jiao Tong University School of Medicine, College of Stomatology, Shanghai Jiao Tong University, National Center for Stomatology, National Clinical Research Center for Oral Diseases, Shanghai Research Institute of Stomatology, Shanghai Key Laboratory of Stomatology, Shanghai, China; ^2^ Hamlyn Centre for Robotic Surgery, Institute of Global Health Innovation, Imperial College London, London, United Kingdom; ^3^ School of Mechanical Engineering, Shanghai Dianji University, Shanghai, China; ^4^ School of Electronic Information and Electrical Engineering, Shanghai Jiao Tong University, Shanghai, China; ^5^ Shanghai Lanhui Medical Technology Co., Ltd., Shanghai, China; ^6^ Faculty of Dentistry, The University of Hong Kong, Hong Kong, Hong Kong SAR, China; ^7^ Department of Radiology, Shanghai Ninth People’s Hospital, College of Stomatology, Shanghai Jiao Tong University School of Medicine, Shanghai, China; ^8^ School of Biomedical Engineering, Shanghai Jiao Tong University, Shanghai, China

**Keywords:** deep learning, craniomaxillofacial surgery, virtual surgical planning, computed tomography, segmentation

## Abstract

**Objectives:**

Accurate segmentation of craniomaxillofacial (CMF) structures and individual teeth is essential for advancing computer-assisted CMF surgery. This study developed CMF-ELSeg, a novel fully automatic multi-structure segmentation model based on deep ensemble learning.

**Methods:**

A total of 143 CMF computed tomography (CT) scans were retrospectively collected and manually annotated by experts for model training and validation. Three 3D U-Net–based deep learning models (V-Net, nnU-Net, and 3D UX-Net) were benchmarked. CMF-ELSeg employed a coarse-to-fine cascaded architecture and an ensemble approach to integrate the strengths of these models. Segmentation performance was evaluated using Dice score and Intersection over Union (IoU) by comparing model predictions to ground truth annotations. Clinical feasibility was assessed through qualitative and quantitative analyses.

**Results:**

In coarse segmentation of the upper skull, mandible, cervical vertebra, and pharyngeal cavity, 3D UX-Net and nnU-Net achieved Dice scores above 0.96 and IoU above 0.93. For fine segmentation and classification of individual teeth, the cascaded 3D UX-Net performed best. CMF-ELSeg improved Dice scores by 3%–5% over individual models for facial soft tissue, upper skull, mandible, cervical vertebra, and pharyngeal cavity segmentation, and maintained high accuracy Dice > 0.94 for most teeth. Clinical evaluation confirmed that CMF-ELSeg performed reliably in patients with skeletal malocclusion, fractures, and fibrous dysplasia.

**Conclusion:**

CMF-ELSeg provides high-precision segmentation of CMF structures and teeth by leveraging multiple models, serving as a practical tool for clinical applications and enhancing patient-specific treatment planning in CMF surgery.

## 1 Introduction

Craniomaxillofacial (CMF) deformities include congenital and acquired malformations such as dentofacial, post-traumatic, post-tumor resection–related, and temporomandibular joint deformities, which significantly compromise the facial aesthetics and stomatognathic functions of patients (Xia et al., 2009). Surgical correction of CMF deformities is challenging due to their complex characteristics. To achieve favorable surgical outcomes, personalized and precise surgical plans are necessary (Alkhayer et al., 2020; On et al., 2024). Recently, virtual surgical planning (VSP) based on three-dimensional (3D) imaging technologies, including 3D preoperative treatment planning and simulation of surgical outcome, has been increasingly utilized in CMF surgery, facilitating deformity diagnosis, cephalometric analysis, surgical simulation, and the fabrication of cutting guides and splints (Naran et al., 2018). The initial step of the VSP workflows involves the segmentation of CMF structures, followed by 3D reconstruction of the composite dental-maxillofacial model from computed tomography (CT) scans (Bao et al., 2024). Overall, efficient and accurate segmentation approaches provide a robust basis for advancing computer-assisted CMF surgery.

Manual segmentation by experienced clinicians acts as the gold standard, but it is widely acknowledged that this process is considerably time-consuming, labor-intensive, and error-prone with segmentation performance varying among experts. In current clinical applications, semi-automatic approaches like threshold-based, region-growing or template-fitting methods (e.g., GrowCut, Canny Segmentation and Robust Statistics Segmenter algorithms) integrate automated segmentation with manual label annotation by experts, which have been applied in digital planning software and alleviate the workload of clinicians (Wallner et al., 2019; Zhang L. et al., 2023). However, instance segmentation, which involves distinguishing and delineating each unique structure within the CMF region, remains challenging due to substantial interindividual morphological variations, intricate structural connections, poor contrast in joints and tooth apices, and frequent presence of artifacts (Priya et al., 2024; Xiang et al., 2024). Traditional approaches still cannot achieve favorable segmentation results and need manual adjustment for clinical use. Therefore, establishing a fully automated, high-precision segmentation system holds considerable clinical significance for CMF surgery.

With the rising clinical needs and the development of artificial intelligence, deep learning has been applied across various aspects of healthcare, including medical diagnosis, treatment planning, surgical assistance, postoperative monitoring and rehabilitation training (Jiang et al., 2017; Chen et al., 2022; Huang et al., 2024; Wang et al., 2024). In the field of dentistry, deep learning has significantly improved digital dentistry workflows such as caries detection, prosthetic evaluation, orthodontic analysis, periodontitis diagnosis and treatment planning (Graves and Uribe, 2024; Nordblom et al., 2024; Setzer et al., 2024). Fully automated medical image segmentation approaches based on deep learning have been proposed to overcome previous limitations and enhance the precision and efficiency of CMF surgery due to its ability to learn features associated with target tasks from large-scale data (Liu et al., 2023; Nogueira-Reis et al., 2023; Xiang et al., 2024). Inspired by its remarkable advancements, many studies have developed and evaluated specific algorithms for CMF CT or Cone-beam CT (CBCT) segmentation (Zhang et al., 2020; Liu et al., 2024). Notably, U-Net-based framework demonstrated excellent performance for medical image segmentation, which has an encoder-decoder framework with skip connections (Ronneberger et al., 2015). Liu et al. proposed a 3D U-Net-based model to segment midface and mandible from CBCT for computer-aided CMF surgical simulation (Liu et al., 2021; Deng et al., 2023). Dot et al. evaluated the performance of the nnU-Net for automatic segmentation of the upper skull, mandible, upper teeth, lower teeth and mandibular canal from CT scans for orthognathic surgery (Dot et al., 2022). However, some limitations restrict their clinical applicability. First, most existing algorithms were dedicated to coarse segmentation considering few structures (e.g., less than 30 structures), and only a few studies have attempted to segment all the structures of interest (facial soft tissue, upper skull, mandible bone, cervical vertebra, hyoid bone, pharyngeal cavity, inferior alveolar nerve, upper teeth, lower teeth and individual teeth), which limits models’ clinical application (Dot et al., 2022; Liu et al., 2024). Second, due to the diverse sizes and shapes of different structures, direct cross-scale training leads to the deficiency of semantic information in multiple segmentation tasks and the capability of the individual model for cross-scale information extraction is limited. The segmentation accuracy and robustness require improvement. To date, no studies have investigated the use of ensemble learning strategies to improve the potential of fully automated segmentation algorithms for application in CMF surgery. Meanwhile, although current studies focusing on segmentation algorithms are promising, the reliability and accessibility of different methods for multi-structure segmentation and classification in the CMF region have not been systematically and comprehensively benchmarked (Schneider et al., 2022). Most published studies were conducted based on small-size hold out dataset (Dot et al., 2024). Hence, it is of great significance to systematically evaluate the segmentation performance of existing models and to develop a novel multi-objective segmentation model capable of automatically extracting information across different scales.

Based on previous studies and the identified deficiencies, the current study aims to comprehensively benchmark the performance of three 3D U-Net-based deep learning models (V-Net, nnU-Net, 3D UX-Net) for multi-structure segmentation and classification using an identical CMF CT dataset. In addition, we propose a novel fully automated framework, named CMF-ELSeg, that utilizes deep ensemble learning methodologies specifically tailored for multi-class segmentation in CMF surgery. By integrating the strengths of each individual model, CMF-ELSeg achieves accurate segmentation of CMF structures and teeth, and can identify each tooth according to the Fédération Dentaire Internationale (FDI) classification. Our framework serves as a powerful tool for surgical planning, significantly enhancing the decision-making and design processes in CMF surgery.

## 2 Materials and methods

**FIGURE 1:**
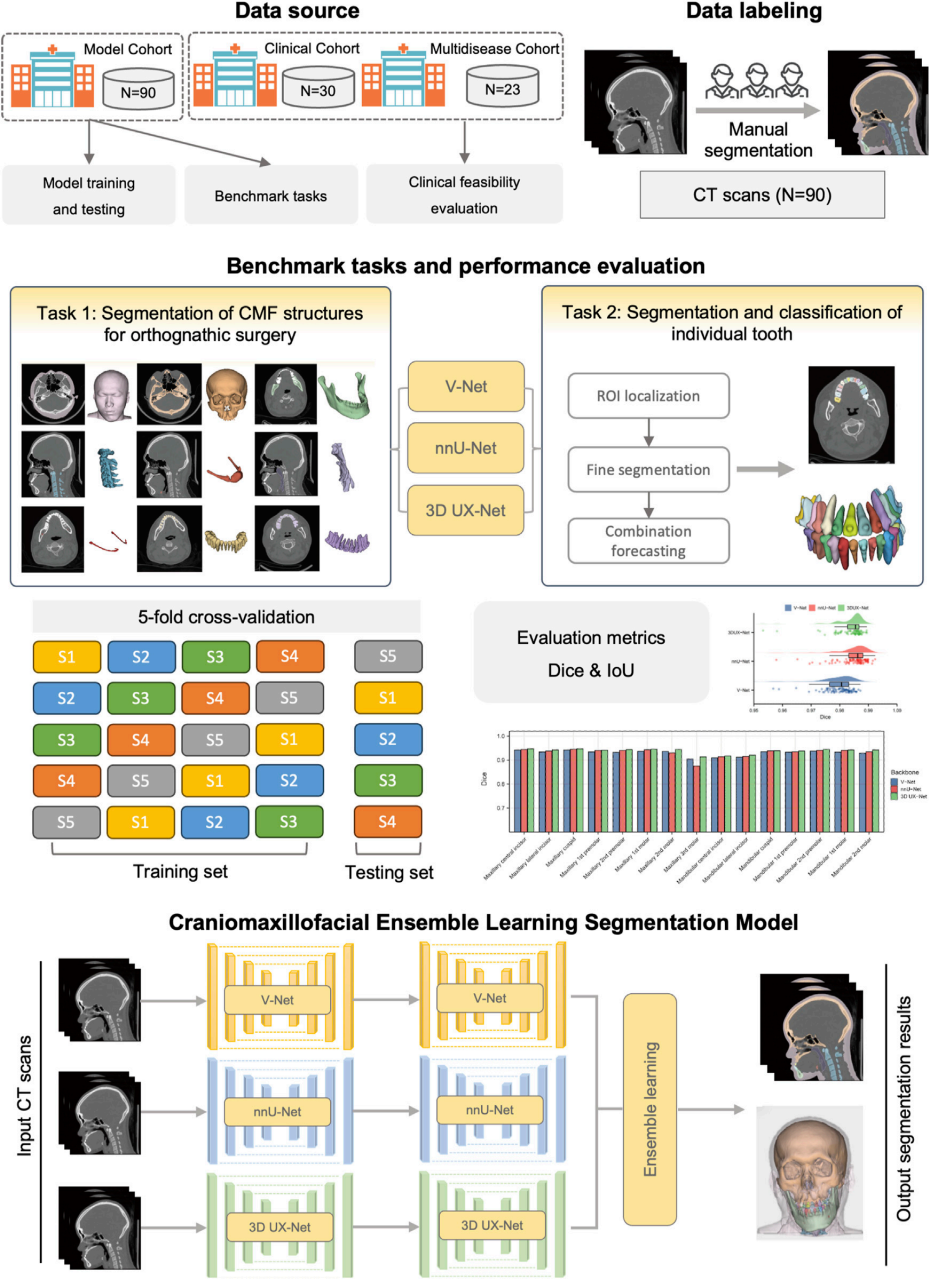
Overview of the study design.

The overview of the study design is shown in Figure 1. Our study follows the Checklist for Artificial Intelligence in Dental Research (Schwendicke et al., 2021). This study was ethically approved by the ethics committee of Shanghai Ninth People’s Hospital, Shanghai Jiao Tong University School of Medicine (IRB No. SH9H-2022-TK12-1).

### 2.1 Participants and dataset

Three cohorts were formed in our study to train and validate the segmentation model. CMF CT scans were collected from the Department of Oral and Cranio-Maxillofacial Surgery, Shanghai Ninth People’s Hospital. Cohort 1 (Model cohort) and Cohort 2 (Clinical cohort) were employed for model training and clinical feasibility evaluation. The inclusion criteria for Cohort 1 and Cohort 2 were as follows: (1) patients diagnosed with skeletal malocclusion; (2) patients who required orthodontic and orthognathic joint treatment; (3) patients who received CMF CT scans covering the entire maxillofacial region. Participants were excluded if they met any of the following conditions: (1) refusal to participate (*n* = 4); (2) inadequate image quality that did not meet the requirements for surgical planning (*n* = 8); (3) diagnosis of congenital dentofacial deformities, such as CMF syndromes, cleft lips, and cleft palates (*n* = 18). Preoperative CT scans were taken during the VSP phase following the completion of preoperative orthodontic treatment, while postoperative CT scans were obtained 6 months after surgery. The parameters of CT images are as follows: a pixel size ranging from 0.40 mm × 0.40 mm to 0.53 mm × 0.53 mm; a slice interval between 0.625 mm and 1.250 mm; and a resolution of 512 × 512 pixels. The details of patient characteristics are shown in the Supplementary Material (Supplementary Figures S1, S2). In addition, the Cohort 3 (Multi-disease cohort), including samples from patients with maxillofacial fractures, fibrous dysplasia, and congenital syndromes, was used to validate the generalizability of the model.

### 2.2 Data annotations

In total, 90 CT scans in Cohort 1 were obtained in Digital Imaging and Communications in Medicine (DICOM) format and imported into 3D Slicer software (version 4.2.0). Manual segmentation for each CT scan was completed by two experienced radiologists and verified by one oral and maxillofacial doctor with rich experience in CMF surgery. The ground truth of each segmentation label was generated, including facial soft tissue, upper skull, mandible bone, cervical vertebra, hyoid bone, pharyngeal cavity, inferior alveolar nerve, upper teeth, lower teeth and individual teeth (Supplementary Figure S3). All ground truth annotations were carefully reviewed to meet the high standards required for clinical use. The details of data annotations and preprocessing were shown in the Supplementary Material.

### 2.3 Benchmark tasks and construction of cascaded segmentation networks

**FIGURE 2:**
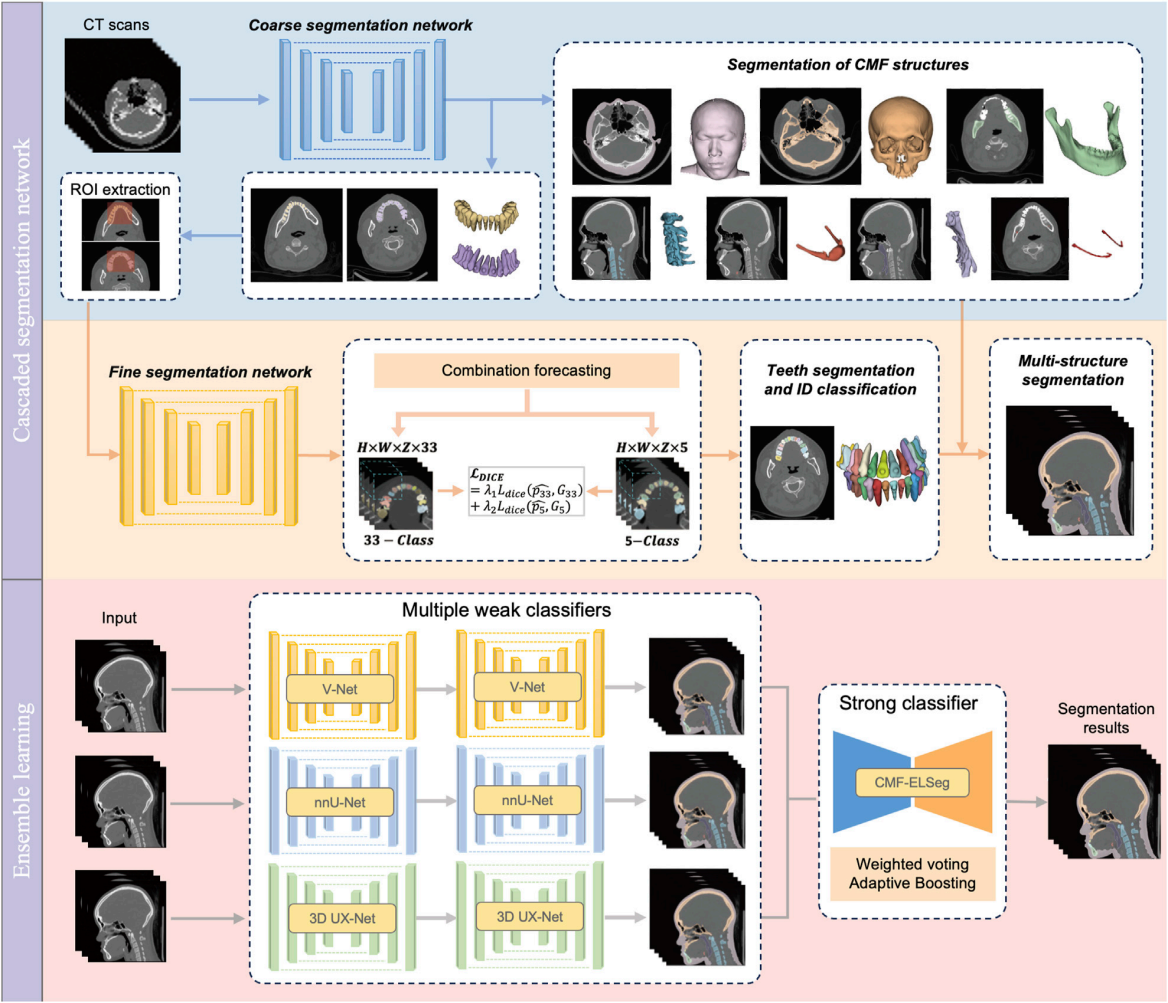
Architecture of our proposed deep learning model.

Our benchmark includes two tasks: (1) comparing the performance of different backbones in CMF structures segmentation; and (2) comparing the performance of fine segmentation networks in teeth instance segmentation. Considering the deficiency of semantic information details in direct cross-scale training, and the difficulty in simultaneously achieving effective recognition of segmentation tasks at different granularities, the cascaded segmentation network illustrated in Figure 2 was developed, which is composed of the coarse segmentation network for CMF structures segmentation and the fine segmentation network for teeth segmentation and ID classification. Three widely used U-Net models including V-Net, nnU-Net, and 3D UX-Net were selected as backbones for training and benchmark evaluation (Milletari et al., 2016; Isensee et al., 2021; Lee et al., 2023). The descriptions of the backbone models are included in the Supplementary Material (Supplementary Figure S4).

In the coarse stage, the CMF anatomical structures of interest (facial soft tissue, upper skull, mandible bone, cervical vertebra, hyoid bone, pharyngeal cavity, inferior alveolar nerves) were first segmented. The teeth are roughly categorized into upper and lower classes according to the position of the teeth in the maxilla and mandible. Then, the CT images and labels were synchronously scaled and cropped using nearest neighbor interpolation based on the foreground region of the upper and lower teeth to obtain the regions of interest (ROI). In the fine stage, the framework of fine segmentation networks shared the same basic architecture as the model from the first stage. A combination forecasting approach based on the features of adjacent teeth to reduce misidentification was applied to achieve fine segmentation of individual teeth. The final layer of the decoder includes two output layers corresponding to the segmentation of teeth into 33 classes (32 individual teeth and the background) and five classes (odd and even-numbered upper and lower teeth, along with the background), respectively. This improvement utilizes a Dice loss function for the five-class segmentation to correct the 33-class segmentation. The loss function for the fine segmentation network is defined as follows:

$$L=\lambda_{1}L_{dice}\left(\hat{p_{33}},G_{33}\right)+\lambda_{2}L_{dice}\left(\hat{p_{5}},G_{5}\right)$$

Where $\hat{p_{33}}$ and $G_{33}$ represent the predicted results and ground truth for 33-class tooth segmentation, respectively, and $\hat{p_{5}}$ and $G_{5}$ represent the predicted results and ground truth for five-class tooth segmentation, respectively. The accuracy of individual cascaded network with different backbones was compared in the task of teeth segmentation.

### 2.4 Framework of CMF-ELSeg

Based on the performance of the coarse-to-fine cascaded segmentation networks, we proposed an ensemble learning segmentation model for CMF surgery, named CMF-ELSeg, to enhance overall segmentation ability (Figure 2). Three cascaded segmentation networks, employing V-Net, nnU-Net, and 3D UX-Net as backbones, were integrated for the development of the ensemble model. Each backbone was chosen for its distinct advantages in voxel recognition. Each individual cascaded segmentation network was trained separately and the CMF-ELSeg was developed leveraging the diversity of different models through a weighted voting strategy to produce a fused segmentation result. To avoid overfitting and poor robustness, we employed ensemble learning to learn the trainable voting weights. Specifically, the Adaptive Boosting (AdaBoost) method was utilized for adaptively assigning appropriate weights to the classifiers during the training process, which is advantageous in effectively reducing bias and variance, thereby improving overall generalization and accuracy (Freund and Schapire, 1997; Schapire, 2013). By combining multiple weak classifiers and iteratively adjusting the weights of the dataset to focus on previously misclassified samples, AdaBoost enhanced the performance of the segmentation task. The weights of weak classifiers were calculated by evaluating the accuracy of three individual cascaded segmentation networks according to the following formula:

$$\alpha_{t}=\frac{1}{2}\ln\left(\frac{1-\epsilon_{t}}{\epsilon_{t}}\right),t=1,2,3$$

where t represents the number of three individual cascaded segmentation network, and $\epsilon_{t}$ represents the error rate of the different model. Subsequently, we integrated multiple weak classifiers to construct a strong classifier, thereby enhancing the accuracy of segmentation tasks.

### 2.5 Evaluation of model performance

Both qualitative and quantitative assessments were conducted. By inputting the original CT images into the deep learning models, we obtained the segmentation results (predictions) for each target. For visualization, individual CMF 3D models were reconstructed employing 3D Slicer software. The segmentation performance of the deep learning models for each CMF structure and individual tooth was assessed by comparing the predictions with manually delineated ground truth. Quantitative evaluation metrics including the Dice and Intersection over Union (IoU) were utilized for this evaluation. The specific definitions of these metrics are listed in the Supplementary Material.

### 2.6 Evaluation of clinical feasibility

Cohort 2 and Cohort 3 were used to validate the clinical feasibility and generalizability of CMF-ELSeg. Specifically, Cohort 2 comprised 30 patients with skeletal malocclusion, while Cohort 3 included 23 patients with a variety of CMF conditions. A four-point categorical scale was used to evaluate the segmentation quality of each category as well as the overall segmentation performance: “Grade A” = optimal automatic segmentation, indicating that the results can be directly used for VSP (The overall grade can only be rated as “A” if all individual categories are also graded as “A”); “Grade B” = minor visual errors in the automatic segmentation, with results still deemed suitable for direct use in surgical planning; “Grade C” = segmentation errors that could impact surgical planning but are easily correctable, such as defects in the anterior wall of the maxillary sinus, misidentification of individual teeth, or discontinuities in the nerve canal; and “Grade D” = significant errors that are difficult to manually correct and adversely affect surgical planning, requiring re-segmentation, such as misidentification of multiple teeth or incorrect classification of the maxilla and mandible (Deng et al., 2023). The segmentation and reconstruction results were evaluated by three experienced surgeons, who collaboratively determined the grade for each label and the overall performance.

Following the qualitative evaluation, the experts responsible for the initial manual annotations refined the preliminary segmentation results rated as B, C, or D. Manual corrections were performed using the 3D Slicer to modify and correct mislabeled regions slice by slice. The revision continued until the segmentation quality for each anatomical structure and individual tooth fully satisfied the criteria of Grade A, indicating that the corrected results could be directly employed for VSP. Dice coefficients were subsequently calculated to compare the automatic segmentation results with the manually corrected outcomes. The corresponding modification times were also recorded throughout this process.

### 2.7 Statistical analysis

All these models underwent training using a 5-fold cross-validation strategy. The analysis and visualization of all data were conducted using Python (v.3.7) and R software (v.4.1.2). For statistical analysis, categorical variables were presented as numbers and percentages, and continuous variables were presented as means ± standard deviations (mean ± Std). We employed the *T*-test for normally distributed continuous variables, and the Mann-Whitney U test for non-normally distributed continuous variables to compare continuous variables between two groups. *P* < 0.05 was considered as the statistical significance.

## 3 Results

### 3.1 Performance evaluation of cascaded segmentation networks for CMF structures and individual teeth segmentation

**FIGURE 3:**
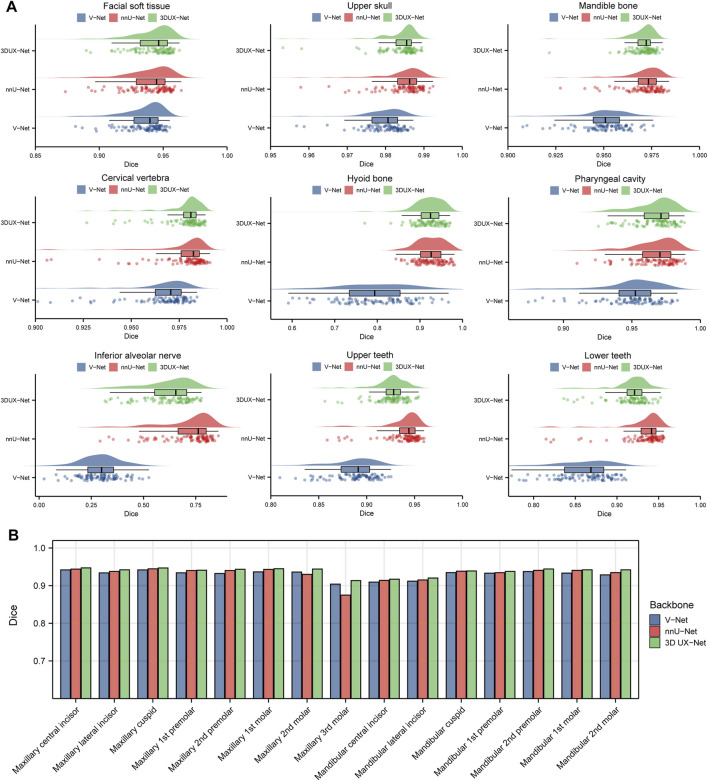
Quantitative analysis results for segmentation performance. **(A)** Dice scores for segmentation performance of CMF structures using V-Net, nnU-Net, and 3D UX-Net. **(B)** Dice scores for segmentation performance of individual teeth using cascaded segmentation networks based on V-Net, nnU-Net, and 3D UX-Net.

Figure 3 and Supplementary Table S2 show the performance (Dice and IoU) of V-Net, nnU-Net, and 3D UX-Net for CMF structures segmentation. The segmentation performance of 3D UX-Net and nnU-Net was comparable and significantly better than that of V-Net (Figure 3A; Supplementary Figure S5A). In the segmentation task for the upper skull, mandible, cervical vertebra and pharyngeal cavity, both 3D UX-Net and nnU-Net achieved average Dice scores exceeding 0.96 and average IoU exceeding 0.93. The nnU-Net generally has the highest mean value on all metrics, particularly excelling in the segmentation of the hyoid bone, inferior alveolar nerve, upper teeth, and lower teeth.

The performance of fine segmentation for individual teeth was evaluated and the quantitative analysis results were presented in Figure 3B and Supplementary Figure S5B. The cascaded segmentation network based on 3D UX-Net demonstrated optimal performance across all evaluation metrics, maintaining high accuracy and stability even when segmenting the maxillary 3rd molar (Dice = 0.9133 ± 0.0778; IoU = 0.8514 ± 0.1153) (Supplementary Table S3). nnU-Net based model’s Dice and IoU scores were slightly lower than those of 3D UX-Net but higher than those of V-Net except for the maxillary 3rd molars. Additionally, the most notable segmentation error made by the model based on nnU-Net was the mislabeling of individual teeth, which occurs less frequently in the model developed based on 3D UX-Net.

### 3.2 Performance evaluation of CMF-ELSeg

**FIGURE 4:**
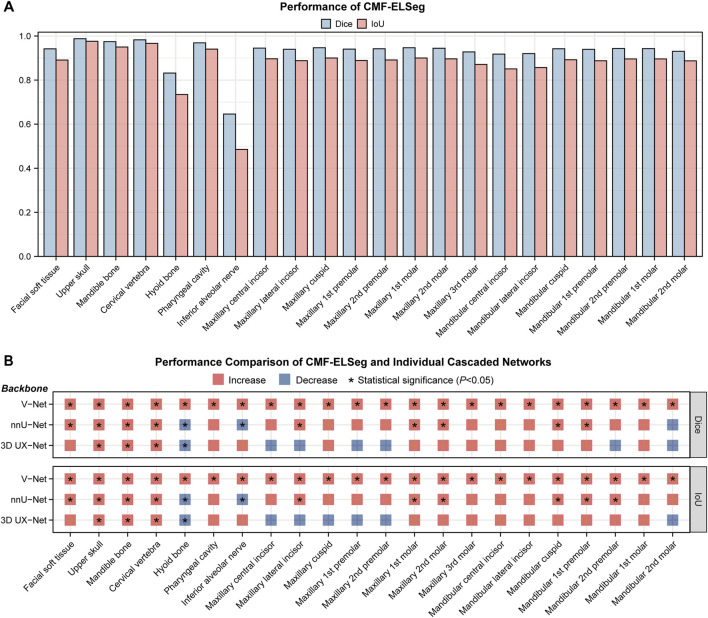
Segmentation performance of CMF-ELSeg. **(A)** Quantitative analysis results for segmentation performance of CMF structures and individual teeth. **(B)** Comparison of segmentation performance between CMF-ELSeg and the baseline models. *P < 0.05.

**FIGURE 5:**
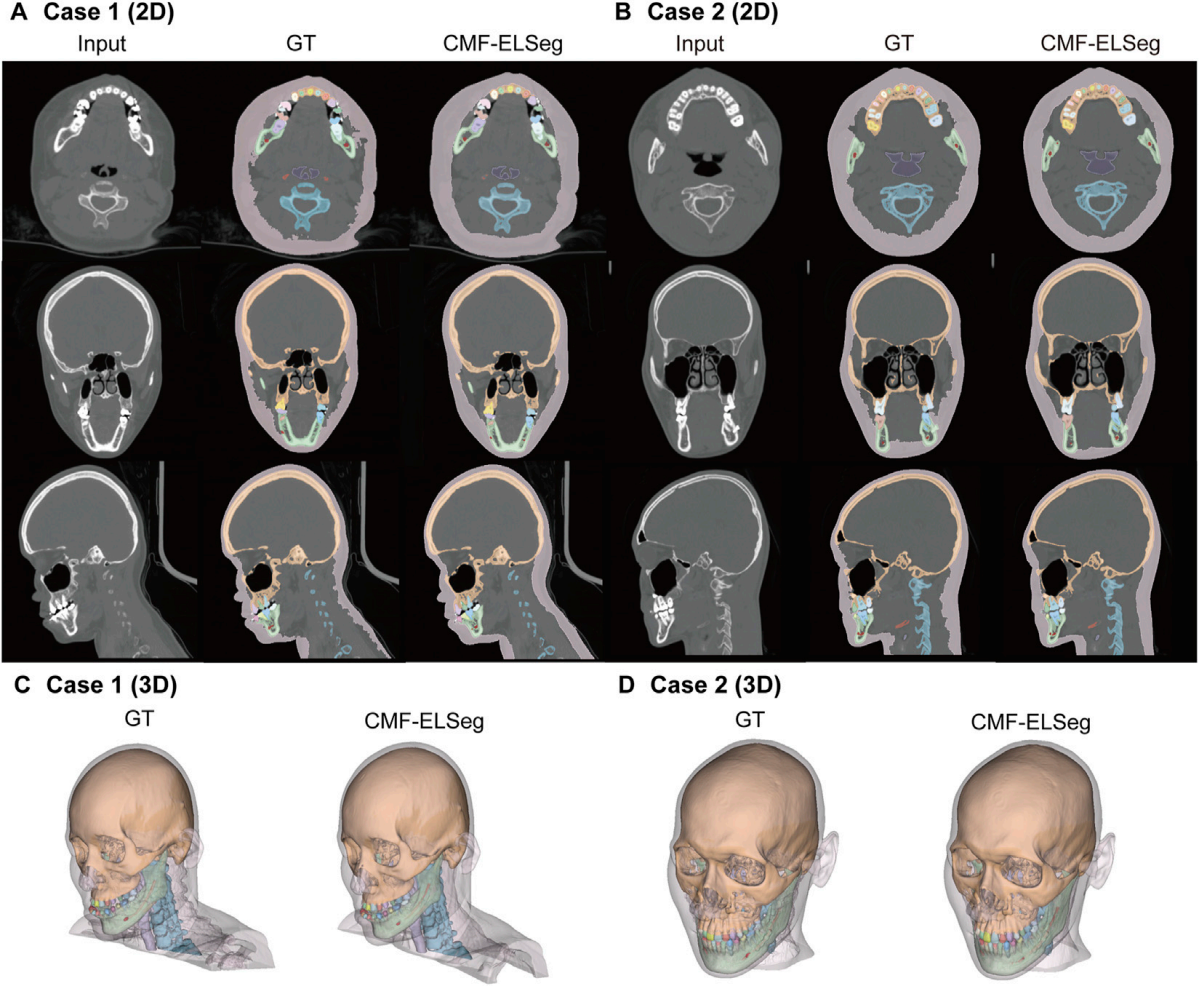
Segmentation and 3D reconstruction results of CMF structures and individual teeth using CMF-ELSeg. **(A,B)** Segmentation results illustrated for two representative cases. **(C,D)** 3D reconstruction results illustrated for two representative cases. Case 1: a skeletal class III malocclusion patient with orthodontic brackets. Case 2: a patient who has undergone orthognathic surgery.

**FIGURE 6:**
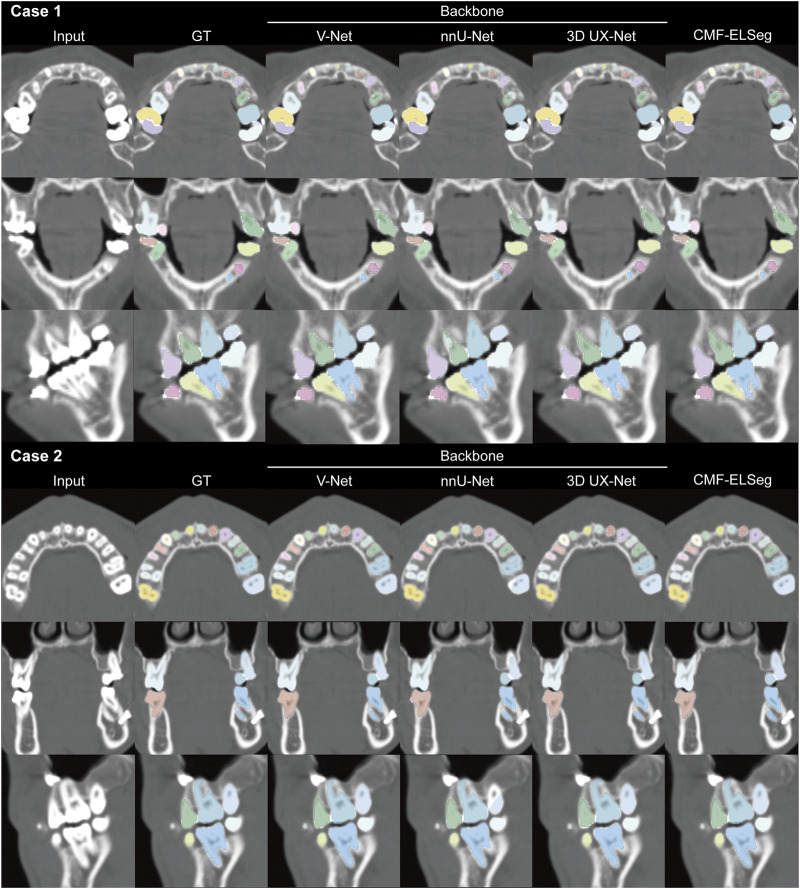
Segmentation results of individual teeth using CMF-ELSeg and individual cascaded segmentation network. Case 1: a skeletal class III malocclusion patient with orthodontic brackets. Case 2: a patient who has undergone orthognathic surgery.

**FIGURE 7:**
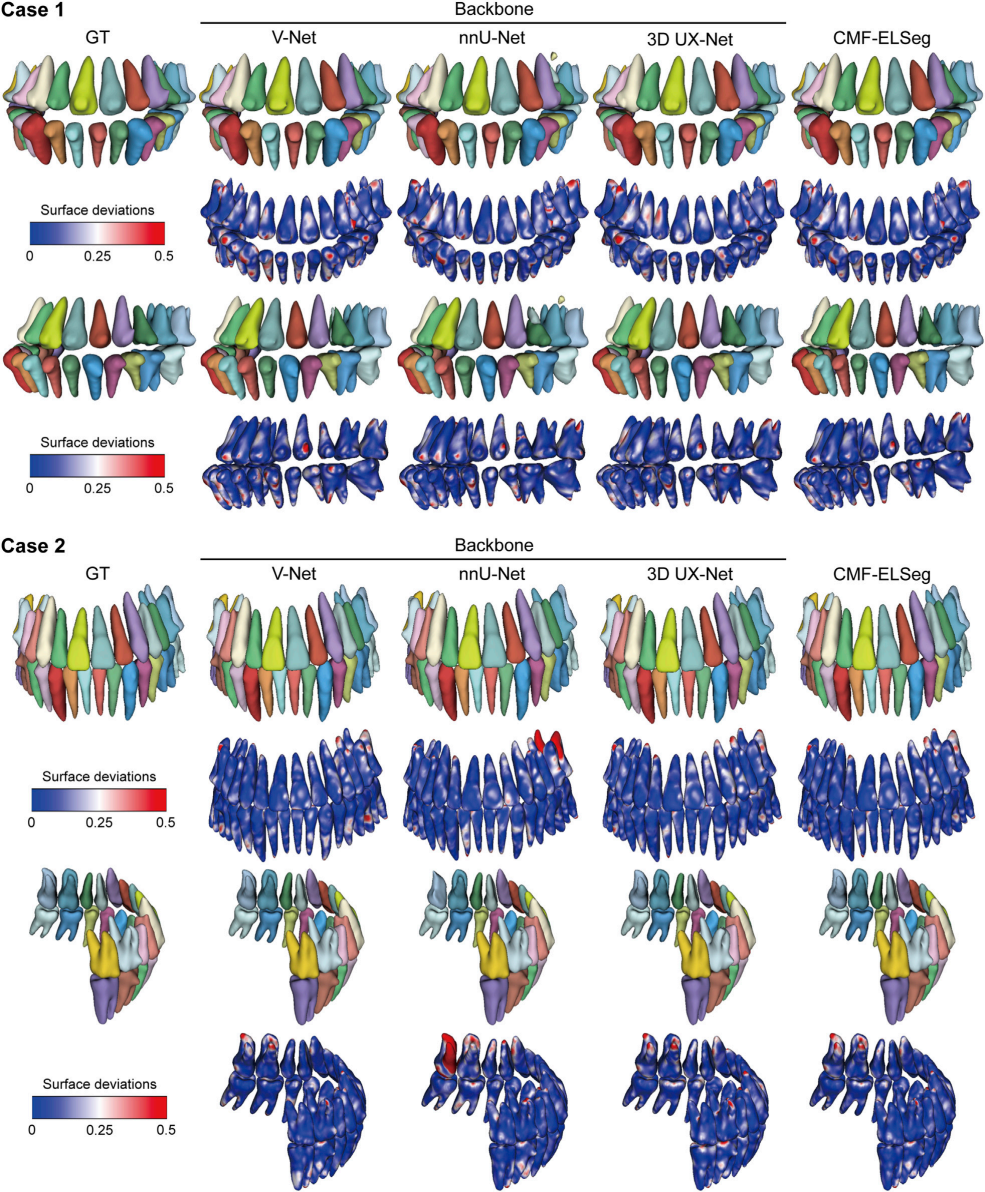
3D reconstruction results and surface deviations of individual teeth using CMF-ELSeg and individual cascaded segmentation network. Case 1: a skeletal class III malocclusion patient with orthodontic brackets. Case 2: a patient who has undergone orthognathic surgery.

The mean results of Dice and IoU for each segmentation label are shown in Figure 4A and Supplementary Table S4. Two cases were randomly selected from our dataset to illustrate our results (Case 1: a patient with dentofacial deformity before orthognathic surgery; Case 2: a patient who has undergone orthognathic surgery). It can be observed that apart from suboptimal segmentation performance for the hyoid bone and inferior alveolar nerves (Dice coefficient less than 0.9), CMF-ELSeg consistently achieves high segmentation levels across other categories. Compared to individual models, CMF-ELSeg demonstrated approximately a 3%–5% improvement in Dice coefficient scores in the segmentation of CMF structures including facial soft tissue, upper skull, mandible bone, cervical vertebra, and pharyngeal cavity (Figure 4B). Figures 5A–D and Supplementary Figure S6 showed the results of 2D segmentation and 3D reconstruction for each label. Additionally, the results of 2D segmentation, 3D reconstruction and surface deviations of all teeth were presented in Figures 6, 7. It showed consistently high segmentation accuracy in the segmentation and classification of individual teeth, where CMF-ELSeg achieved Dice exceeding 0.94 for most teeth segmentation tasks, with slightly lower Dice scores observed for Maxillary 3rd molar (0.9282 ± 0.0515), Mandibular central incisor (0.9180 ± 0.0425), Mandibular lateral incisor (0.9204 ± 0.0577), Mandibular 1st premolar (0.9397 ± 0.0332), and Mandibular 2nd molar (0.9307 ± 0.1053) (Supplementary Tables S4, S5).

### 3.3 Clinical feasibility evaluation of CMF-ELSeg

**FIGURE 8:**
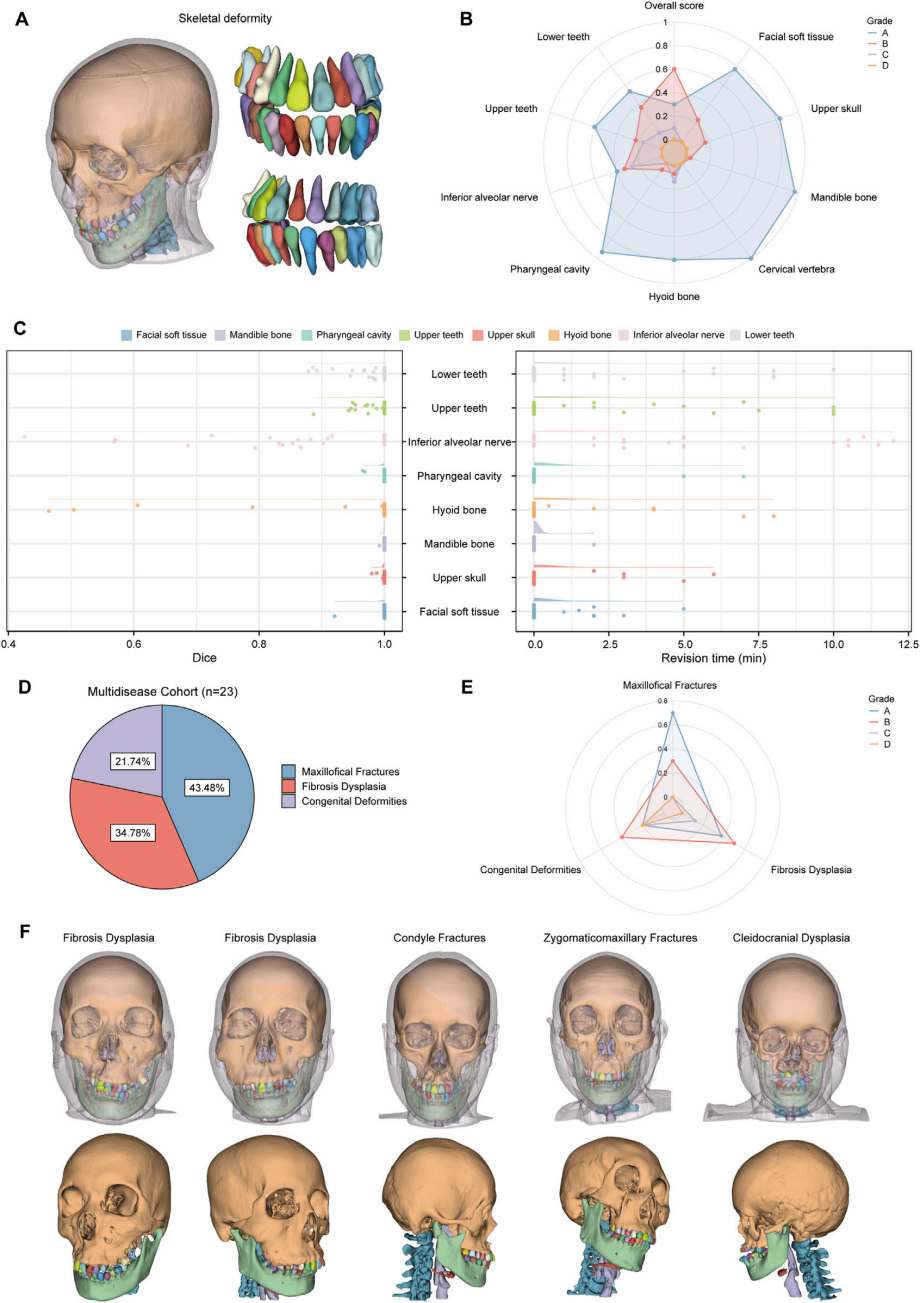
Clinical feasibility evaluation of CMF-ELSeg. **(A)** An example of the segmentation and reconstruction results using CMF-ELSeg for patients with skeletal malocclusion. **(B)** The qualitative analysis results of CMF-ELSeg in Cohort 2. **(C)** Quantitative analysis results of CMF-ELSeg in Cohort 2. **(D)** The composition of patients in Cohort 3. **(E)** The qualitative analysis results of CMF-ELSeg in Cohort 3. **(F)** Segmentation and reconstruction cases of CMF-ELSeg in Cohort 3.

Cohort 2 included 30 patients with skeletal malocclusion. The example of segmentation and the results of the qualitative evaluation of CMF-ELSeg are shown in Figures 8A,B. Among 30 cases, 90% were ranked “Grade A” or “Grade B,” indicating that these results could be directly used for VSP without the need for manual revision. Only 10% of the cases were rated as “Grade C,” with no segmentation results rated as “Grade D.” The quantitative analysis results showed that, except for the segmentation of the hyoid bone (0.943 ± 0.148) and inferior alveolar nerve (0.882 ± 0.153), the average Dice scores for the other structures exceeded 0.975 (Supplementary Table S6; Figure 8C). The revision times were recorded in Supplementary Table S6 and Figure 8C, where the overall revision time was 15.119 ± 10.155 min. Cohort 3 consisted of 23 patients with various craniofacial disorders, including 10 cases of maxillofacial fractures, eight cases of fibrous dysplasia, and five cases of complex craniofacial conditions (cleidocranial dysplasia, secondary deformities from cleft lip and palate) (Figure 8D). CMF-ELSeg demonstrated strong performance in the segmentation and reconstruction of maxillofacial fractures and fibrous dysplasia, with the evaluation of Grade B and above reaching 100% for facial fractures and 87.5% for fibrous dysplasia (Figure 8E). However, the model’s performance significantly decreased when applied to complex craniofacial conditions, with 40% of cases rated as C and D (Figure 8E). The automatic segmentation results are shown in Figure 8F.

## 4 Discussion

Segmentation and reconstruction of CMF structures and individual teeth are essential steps for orthodontics and orthognathic treatment planning. Developing and validating fully automatic segmentation algorithms and selecting the optimal model are of great significance (Zhang R. et al., 2023; Chen et al., 2024). In this study, we designed a novel coarse-to-fine cascaded segmentation network and employed a combination forecasting method to enhance the accuracy of individual teeth segmentation. By comparing three network backbones and utilizing ensemble learning, CMF-ELSeg achieved a 3%–5% improvement in segmentation performance for CMF structures and individual teeth compared to individual models.

To our knowledge, this is the first study to simultaneously segment multiple CMF structures and individual teeth (Cui et al., 2022; Xiang et al., 2024). First, we evaluated the segmentation performance of the three models on nine CMF structures, which are commonly involved in surgical planning. We selected V-Net, nnU-Net, and 3D UX-Net for their complementary strengths in handling complex CMF segmentation needs. Specifically, V-Net’s residual convolutional architecture allows for enhanced feature extraction in volumetric data, while nnU-Net’s self-configuring capabilities make it particularly effective across variable anatomical regions. Meanwhile, 3D UX-Net’s transformer-based architecture captures both global and local features, contributing to high-precision segmentation of individual teeth and other small structures. The ensemble approach leverages these unique strengths, optimizing the performance of CMF-ELSeg in the context of intricate CMF anatomy. The use of Dice and IoU scores across CMF and dental structures provides a robust, multifaceted evaluation of CMF-ELSeg’s segmentation performance. However, the segmentation of the inferior alveolar nerve yielded lower Dice scores, due to its small size and complex trajectory (Ntovas et al., 2024). nnU-Net significantly outperformed the other two models in segmenting elongated anatomical structures, including inferior alveolar nerves and the hyoid bone, making it the preferred choice for its user-friendly features (Isensee et al., 2021). Our findings are consistent with those from Dot et al., who employed the nnU-Net to segment CMF structures from CT scans and demonstrated its reliable performance in accomplishing fully automated segmentation for skeletal malocclusion patients before orthognathic surgery (Dot et al., 2022).

Specifically, due to the lack of semantic detail in direct cross-scale training and the challenge of recognizing segmentation tasks at varying granularities, we constructed a coarse-to-fine cascaded framework for individual tooth segmentation and identification (ID) classification. Among the three backbones, the 3D UX-Net-based model demonstrated the best tooth segmentation capability. By extracting the ROI during the coarse segmentation stage, the model could capture relevant spatial features and attenuate background noise (Jing et al., 2018; Lee et al., 2022). Meanwhile, the feature combination approach effectively addressed the issue of misidentification caused by tooth contact. As our training data were obtained from the VSP stage either before orthognathic surgery or 6 months post-surgery, premolars were often absent. Our experimental results also demonstrated the robust performance of the proposed model when dealing with samples that had missing teeth or significant anatomical and positional variations in wisdom teeth (Zhou et al., 2024). By integrating the multiple CMF structures and individual teeth, the reconstructed 3D models can meet the needs of orthognathic surgery and orthodontic treatment planning.

Another key contribution of our study is the introduction of ensemble learning to CMF surgery, a paradigm in machine learning that enhances methodological performance. Recently, several new segmentation algorithms based on the U-Net architecture have been developed. We selected three U-shaped models as backbones. V-Net extends U-Net from 2D to 3D, enhancing local feature extraction through its residual architecture in each convolutional stage (Milletari et al., 2016). Chen et al. proposed a multi-task method based on the V-Net that can segment different kinds of teeth and deal with non-open bite regions and metal artifacts from CBCT (Chen et al., 2020). nnU-Net integrates multiple U-Net methods such as 2D U-Net and 3D U-Net (Isensee et al., 2021). As a publicly available and user-friendly tool, it can automatically configure itself and adapt to any new dataset without manual intervention. The Vision Transformer (ViT) excels in medical imaging tasks, with some researchers combining it with U-Net to enhance segmentation performance (Berroukham et al., 2023). Jin et al. proposed a novel Swin Transformer–U-Net model to segment and classify nasal and pharyngeal airway subregions (Jin et al., 2023). Compared to CNNs, which focus solely on local image structures, ViT captures global features by analyzing connections between localized regions but has limitations in feature localization. Therefore, some hybrid frameworks combine the complementary strengths of ViT and CNNs. 3D UX-Net, developed by Lee et al., is a U-shaped network combining convolution with Swin Transformer for volumetric segmentation, effectively reducing parameters through its lightweight volumetric ConvNet (Lee et al., 2023). While it has demonstrated state-of-the-art performance in various datasets, its application in CMF structure and tooth segmentation remains unexplored. Here, the proposed ensemble model (CMF-ELSeg) combines multiple cascaded segmentation networks, leveraging their diversity to enhance overall performance (Wang et al., 2023; Roshan et al., 2024). The AdaBoost method effectively reduces bias and variance, improving generalization and accuracy. Experimental results show that CMF-ELSeg significantly outperformed individual cascaded segmentation models. Additionally, our model’s performance in tooth segmentation can be extended to various clinical scenarios, such as orthodontic treatment planning, management of periodontitis patients, and implant restoration design (Polizzi et al., 2023; Polizzi et al., 2024).

This study has several limitations that should be addressed in future work. First, model development and evaluation were conducted using a single-center dataset, necessitating validation through large-sample, multicenter studies. The CT scans were primarily from patients with CMF deformities requiring combined orthodontic and orthognathic treatment. To enhance the model’s applicability and robustness, future research will include a broader patient population, encompassing individuals with complex craniofacial conditions such as fractures, jaw defects, craniofacial syndromes, and cleft lip and palate. Second, our results indicated relatively lower segmentation accuracy for tubular and thin anatomical structures, such as the hyoid bone, inferior alveolar nerve, orbital walls and maxillary sinuses. Addressing these challenges might involve integrating higher-resolution sub-volume inputs specifically focused on these fine structures to enhance spatial resolution. Specialized segmentation architectures, potentially incorporating attention mechanisms or transformer-based modules optimized for thin and tubular structures, could further improve performance. Third, clinical validation revealed poor performance in complex craniofacial conditions like cleft lip and palate and congenital syndromes, where segmentation was compromised. Calcified lesions, such as those in fibrous dysplasia, impacted precision due to varying degrees of calcification. To address these issues, we plan to refine the model by developing specialized algorithms tailored to complex craniofacial conditions and calcified tissues. This will enhance the model’s robustness and applicability across a broader range of clinical cases. In addition, while the cascaded architecture and ensemble inference of CMF-ELSeg significantly enhance segmentation accuracy and robustness, these strategies inherently increase computational complexity and inference time. Although our current inference speed remains clinically acceptable for routine preoperative surgical planning, real-time deployment or integration into interactive clinical workflows may necessitate further optimization. We have developed the VSP-AI platform and integrated our segmentation algorithm into it (Supplementary Figure S7). This platform streamlines the VSP design process, optimizing workflow and improving efficiency. In the future, we plan to conduct clinical validation studies to evaluate the model’s accuracy and efficiency in real-world trials, providing valuable insights into its practical applicability.

## 5 Conclusion

In conclusion, our study introduced CMF-ELSeg, a fully multi-structure segmentation model designed to simultaneously segment multiple CMF structures and individual teeth for orthognathic surgical planning. Built on a coarse-to-fine cascaded segmentation network architecture, CMF-ELSeg leverages an ensemble learning approach that combines the strengths of V-Net, nnU-Net, and 3D UX-Net. This multi-model approach led to a 3%–5% improvement in Dice coefficients for segmentation of facial soft tissue, upper skull, mandible bone, cervical vertebra, and pharyngeal cavity compared to individual models. Additionally, CMF-ELSeg consistently achieved high accuracy for individual teeth segmentation, with Dice coefficients exceeding 0.94 for most teeth. These results underscore CMF-ELSeg’s high precision and its potential as a practical tool for clinical practice, significantly enhancing the efficacy of patient-specific treatment planning for CMF surgery.

## Data Availability

The raw data supporting the conclusions of this article will be made available by the authors, without undue reservation.
