# Supplementary material for: Deep ensemble learning-driven fully automated multi-structure segmentation for precision craniomaxillofacial surgery
Source: Front Bioeng Biotechnol. 2025 May 8;13:1580502. doi: 10.3389/fbioe.2025.1580502 (PMC12094958; doi:10.3389/fbioe.2025.1580502)
Supplement: Supplementary file 1 [file DataSheet1.docx]

Supplementary Material

# Supplementary data

**Dataset construction and patient characteristics**

In Cohort 1, 90 CMF CT scans were obtained from patients (53 females, 22.51±3.98 y; 37 males, 22.08±3.01y) (**Figure S1A**). Most CT scans were obtained using the PHILIPS Brilliance 64 (n=63) and PHILIPS iCT 256 (n=22), and a minor subset of the CT scans was acquired using SIEMENS SOMATOM Definition Flash (n=4) and GE Discovery CT750 HD (n=1). 77 CT scans were taken during the preoperative VSP stage, and 13 scans were taken at the 6-month postoperative follow-up. Our dataset demonstrated various types of deformities (**Figure S1B**). 26 subjects (28.9%) showed skeletal class I (0 ≤ ANB < 4), 52 subjects (57.8%) showed skeletal class III (ANB < 0), 12 subjects (13.3%) showed skeletal class II and 37 subjects (41.1%) exhibited facial asymmetry. Due to the presence of orthodontic materials, metallic crowns, and dental implants, 47 CT scans (52.2%) exhibited metal artifacts (**Figure S1C**). Most patients’ mandibular wisdom teeth were removed, while nearly half have retained their maxillary wisdom teeth, since these can be extracted concurrently during the surgery (**Figure S1D**). Meanwhile, some patients had certain premolars extracted during the preoperative orthodontic phase, while the absence of anterior teeth and molars is often due to congenital or acquired factors. ﻿**Figure S1E** shows the data distribution and **Table S1** summarizes patient characteristics in our datasets. Cohort 2 included 30 patients with skeletal malocclusion. **Figure S2** showed the detailed information of clinical characteristics of patients in the Cohort 2.

**Data annotations**

A total of 90 CT scans from Cohort 1 were obtained in Digital Imaging and Communications in Medicine (DICOM) format and imported into 3D Slicer software (version 4.2.0). Fully manual segmentation of each CT scan was carried out by two experienced radiologists and verified by an oral and maxillofacial surgeon with extensive experience in craniofacial and maxillofacial (CMF) surgery. Experts used tools, such as the brush tool in 3D Slicer, to delineate and refine specific anatomical structures in the sagittal, coronal, and axial views of the CT images. All segmentations were carefully reviewed and refined slice-by-slice to ensure they met the high standards required for clinical use. The ground truth segmentations were generated for each label, including facial soft tissue, upper skull, mandible bone, cervical vertebra, hyoid bone, pharyngeal cavity, inferior alveolar nerve, upper teeth, lower teeth, and individual teeth (**Figure S3**). To evaluate intra- and inter-examiner reliability, 20 CMF CT scans were randomly selected and manually labeled, with a minimum interval of two weeks between labeling sessions. Kappa values were calculated to assess both intra- and inter-class reliability. In cases of disagreement, the experts held discussions to reach a consensus decision.

**Data preprocessing**

For image preprocessing, foreground cropping and a negative-positive ratio method were employed to ensure balanced representation of both classes in the dataset. Specifically, foreground cropping was conducted to isolate relevant regions by identifying foreground pixel values and removing redundant backgrounds. Then, the images were cropped into 8 segments with a 1:1 ratio of negative-to-positive samples. Additionally, random nonlinear transformations were applied to enhance image contrast and reduce redundancy. Each pixel’s intensity underwent a random function mapping, altering the grayscale histogram. These approaches augmented image diversity while minimizing redundant information, thereby optimizing the model’s performance in analyzing CT images.

**Backbone architectures**

To determine the most effective core network for integration into our framework, evaluations were conducted on the performance of V-Net, nnU-Net, and 3D UX-Net (**Figure S4**).

1. **V-Net**

V-Net is a three-dimensional fully convolutional neural network based on the U-Net architecture, consisting of an encoder-decoder structure(Milletari et al., 2016). In the encoder, it is divided into five stages, each containing one to three convolutional layers, with the last three stages forming a 3D convolution block (**Figure S4A**). Each convolutional layer in the stages uses a 5×5×5 kernel with a stride of 1. The input CT image resolution is reduced from $H\times W\times Z\times1$ to $\frac{H}{2}\times\frac{W}{2}\times\frac{Z}{2}$, $\frac{H}{4}\times\frac{W}{4}\times\frac{Z}{4}$, $\frac{H}{8}\times\frac{W}{8}\times\frac{Z}{8}$ and $\frac{H}{16}\times\frac{W}{16}\times\frac{Z}{16}$, through 2×2×2 convolutional layers. As the resolution decreases in the encoder stages, the number of feature channels doubles in each stage. The ReLU non-linear activation function is applied throughout the network to enhance its fitting capability. In the decoder, V-Net extracts features and expands the spatial support of low-resolution feature maps to collect and connect the necessary information for generating two-channel volume segmentation. Using a 1×1×1 convolutional kernel, two feature maps are computed, generating an output with the same size as the input volume. The segmentation probability is predicted using the softmax activation function.

1. **nnU-Net**

nnU-Net is based on the basic architecture of 2D U-Net, 3D U-Net, and U-Net Cascade, focusing on optimizing the automatic training pipelines of these three models to improve model training efficiency (**Figure S4B**)(Isensee et al., 2021). Considering the processing of three-dimensional CT image data, this study chose to focus on 3D U-Net, which is specialized in handling three-dimensional image data, due to its wider applicability. 3D U-Net consists of an encoder-decoder structure. In the encoder, each layer contains two convolutional operations with a kernel size of 3×3×3 and a stride of 1, followed by a ReLU activation function, and then a maximum pooling layer with a kernel size of 2×2×2 and a stride of 2. In the decoder, each layer consists of upsampling operations with a kernel size of 2×2 and a stride of 2, followed by two convolutional operations with a kernel size of 3×3×3 and a stride of 1, and then ReLU activation function. Each stage of the encoder provides high-resolution features required by the decoder through skip connections. In the last layer of the network, the number of output channels is adjusted to the desired quantity by convolutional operations with a kernel size of 1×1×1, and the segmentation probability is predicted by the softmax activation function.

1. **3D UX-Net**

The 3D UX-Net architecture is an enhancement of the 3D U-Net framework, comprising an encoder-decoder structure and incorporating long skip connections to facilitate improved spatial detail recovery (**Figure S4C**) (Lee et al., 2023). In the encoder, a large convolution is applied to the input part ($H\times W\times Z\times1$) to map preprocessed CT images to a low-dimensional spatial feature representation. The resulting feature maps are of size $\frac{H}{2}\times\frac{W}{2}\times\frac{Z}{2}$ and projected into a 48-dimensional space. Within the 3D UX-Net Block, large kernel convolutional layers are utilized to compute the aforementioned feature maps. To accommodate the computation of local self-attention features, the network employs depthwise convolutions of size 7×7×7 with padding of 3, serving as a "shifted window" to evenly partition the feature maps. Subsequently, depthwise convolutional scaling (DCS) layers follow the DWC layers. DCS layers scale the dimensions of feature maps without increasing model parameters, thereby reducing redundant information in cross-channel learning.

In a multiscale environment, this hierarchical feature representation is extracted and utilized for dense volume segmentation, aiding in discerning complex structures within the data. The multiscale outputs of the encoder are connected to the decoder based on CNNs via long skip connections to enhance spatial detail recovery. From each encoder stage, 3D UX-Net extracts output feature maps and stabilizes them through instance normalization using residual blocks consisting of two normalized 3×3×3 convolutional layers. Subsequently, the processed features of each stage undergo upsampling via transpose convolutional layers and are concatenated with features from the previous stage. For downstream volume segmentation tasks, 3D UX-Net connects residual features from input patches with upsampled features and feeds them into residual blocks containing 1×1×1 convolutional layers with softmax activation functions for predicting segmentation probabilities.

**Evaluation of model performance**

Quantitative evaluation metrics including the Dice and Intersection over Union (IoU) were utilized for this evaluation. The specific definitions of these metrics are listed below: Dice measures the pair-wise overlap ratio between the predictions and the ground truth. It ranges from 0 to 1, where 1 indicates perfect agreement. The formula for Dice is given by:

$$Dice (y_{\text{gt}} , y_{\text{pred}})=\frac{2\left| y_{\text{gt}}\cap y_{\text{pred}} \right|}{\left| y_{\text{gt}} \right|+\left| y_{\text{pred}} \right|}$$

where$y_{\text{gt}}$is the ground truth and $y_{\text{pred}}$ is the prediction.

IoU, or Jaccard Index, reflects the overlap ratio between the intersection and union of predictions and the ground truth. It ranges from 0 to 1, with 1 indicating perfect overlap. IoU is defined as:

$$IoU(y_{\text{gt}} , y_{\text{pred}})=\frac{\left| y_{\text{gt}}\cap y_{\text{pred}} \right|}{\left| y_{\text{gt}}\cup y_{\text{pred}} \right|}$$

where$y_{\text{gt}}$is the ground truth and $y_{\text{pred}}$ is the prediction.

**Training details**

The deep learning framework was implemented using the PyTorch, with the Adam optimizer utilized for minimizing loss functions and optimizing network parameters through backpropagation. A learning rate of 0.001 and a mini-batch size of 4 were employed in both the V-Net and 3D UX-Net segmentation networks. During training, the loss on the validation dataset was computed at the end of each epoch to assess network convergence. We trained our model for a maximum of 300 epochs. If the model’s performance on the validation dataset remained stable for 5 consecutive epochs, training was considered to have converged and was halted early. All deep neural networks were trained using one NVIDIA A100 Tensor Core GPU.

**Reference**

Isensee, F., Jaeger, P. F., Kohl, S. A. A., Petersen, J., and Maier-Hein, K. H. (2021). nnU-Net: a self-configuring method for deep learning-based biomedical image segmentation. *Nat Methods* 18, 203–211. doi: 10.1038/s41592-020-01008-z

Lee, H. H., Bao, S., Huo, Y., and Landman, B. A. (2023). 3D UX-Net: A Large Kernel Volumetric ConvNet Modernizing Hierarchical Transformer for Medical Image Segmentation. doi: 10.48550/arXiv.2209.15076

Milletari, F., Navab, N., and Ahmadi, S.-A. (2016). V-Net: Fully Convolutional Neural Networks for Volumetric Medical Image Segmentation. doi: 10.48550/arXiv.1606.04797

# Supplementary Figures and Tables

## Supplementary Figures


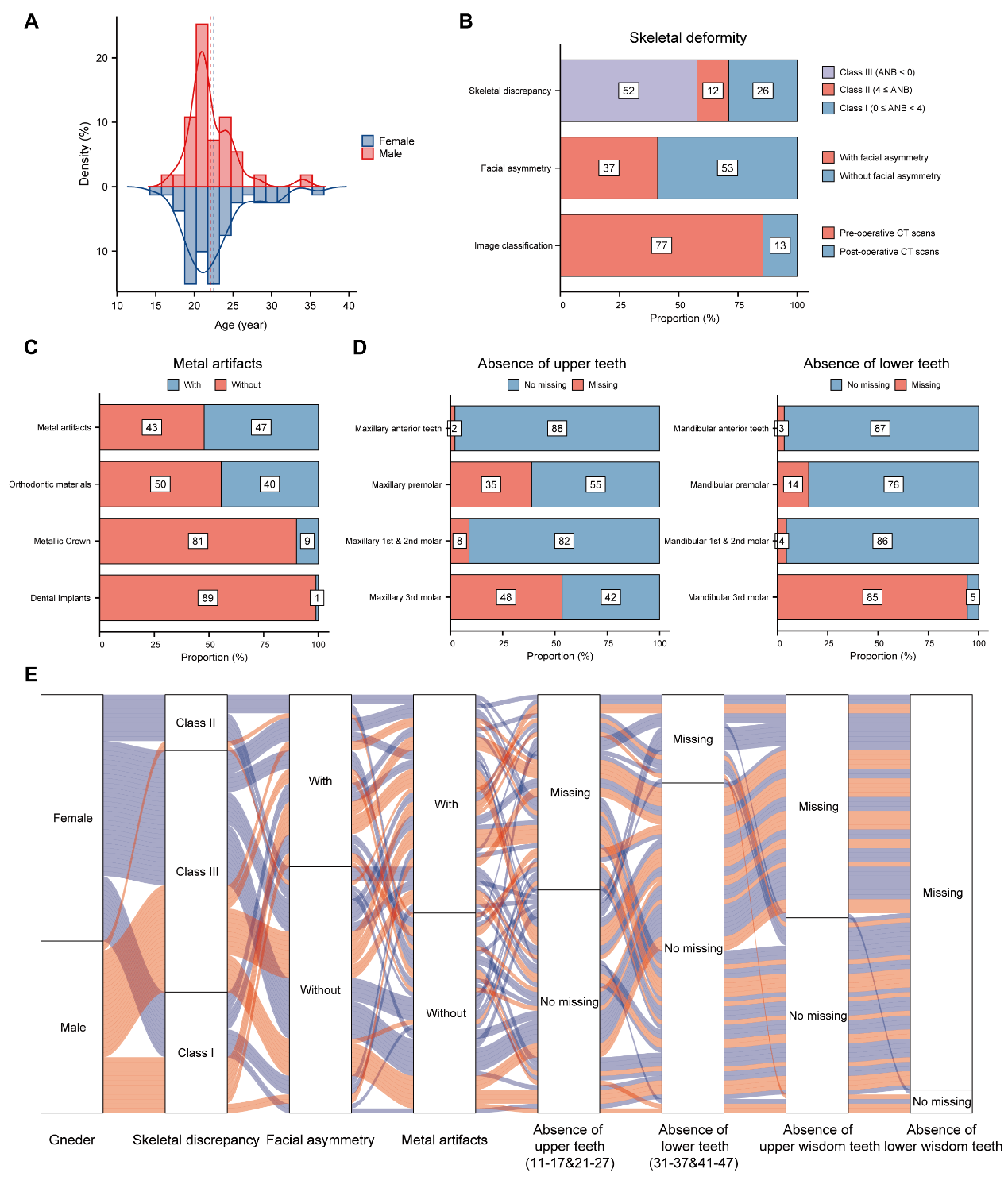


**Figure S1.** Clinical characteristics of patients in the Cohort 1. (A) Age and gender. (B) Skeletal deformity. (C) Metal artifacts. (D) Absence of teeth. (E) The distribution of data.


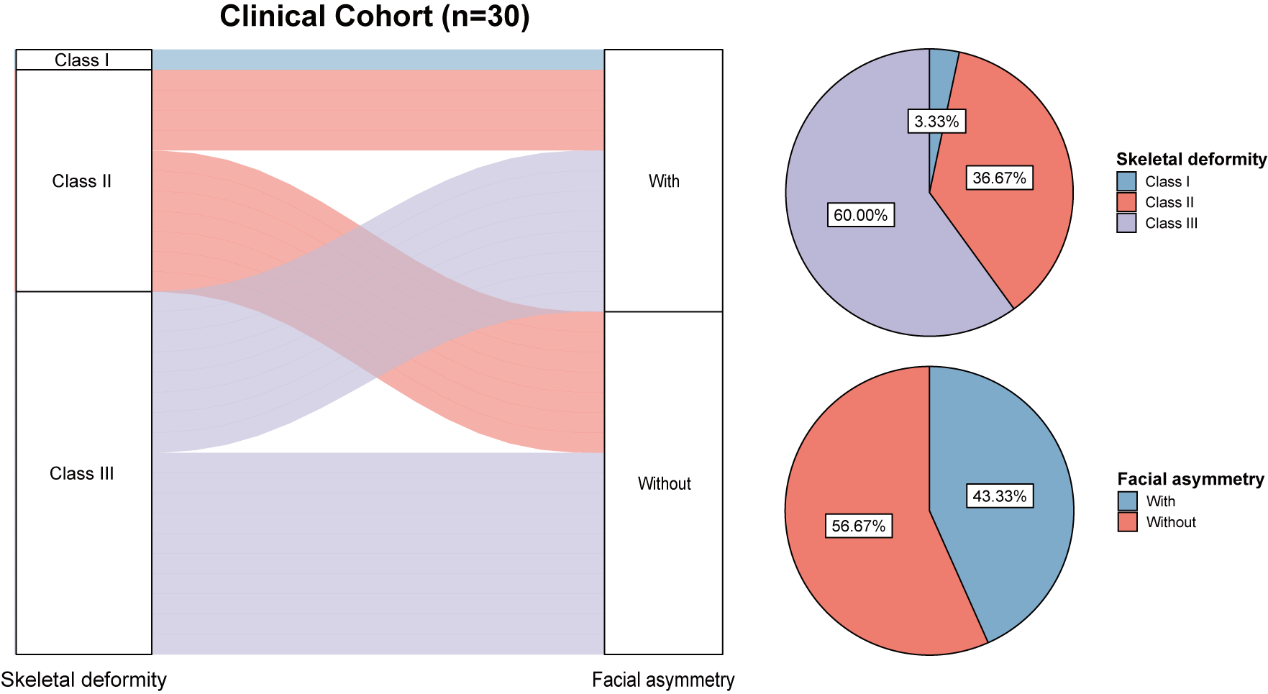


**Figure S2.** Clinical characteristics of patients in the Cohort 2.


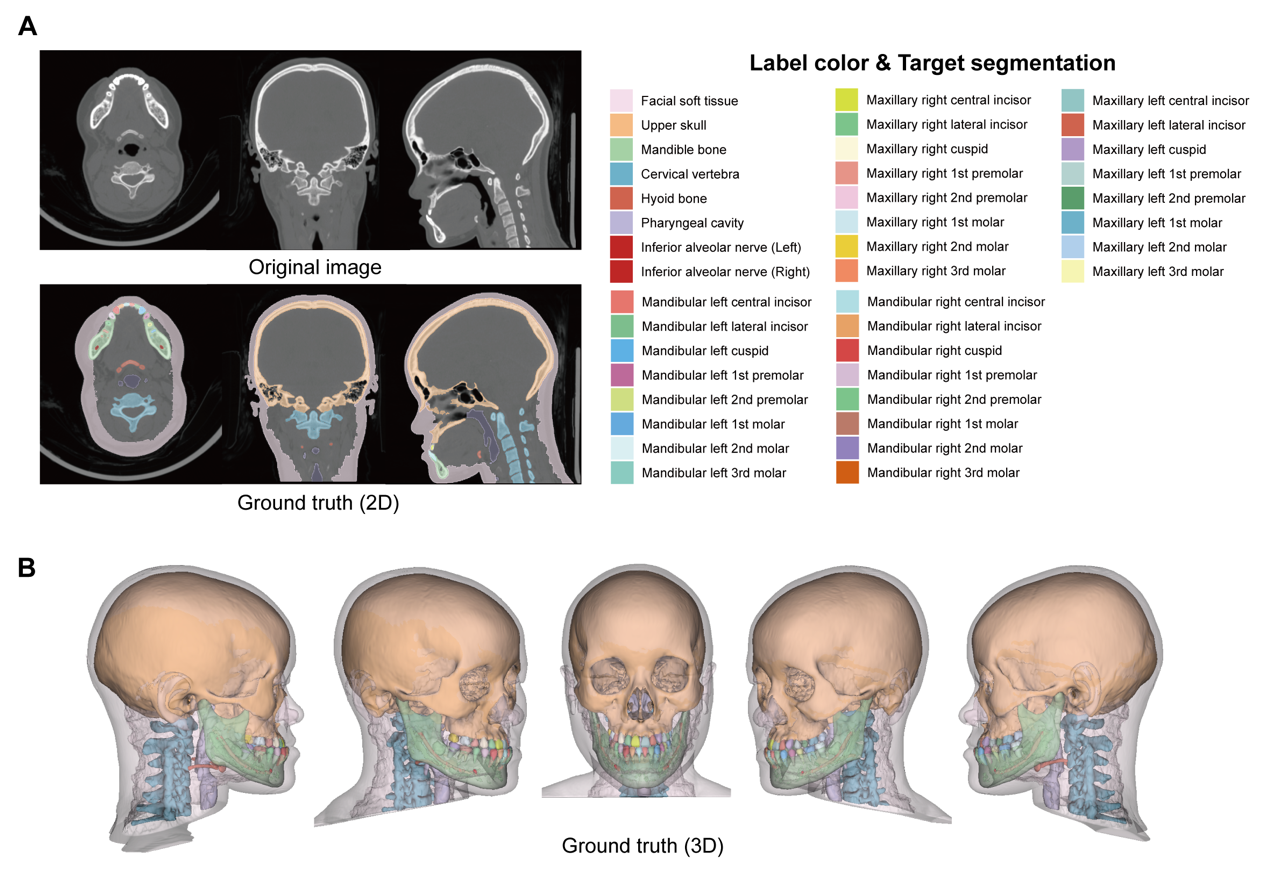


**Figure S3**. Segmentation labels in our study.


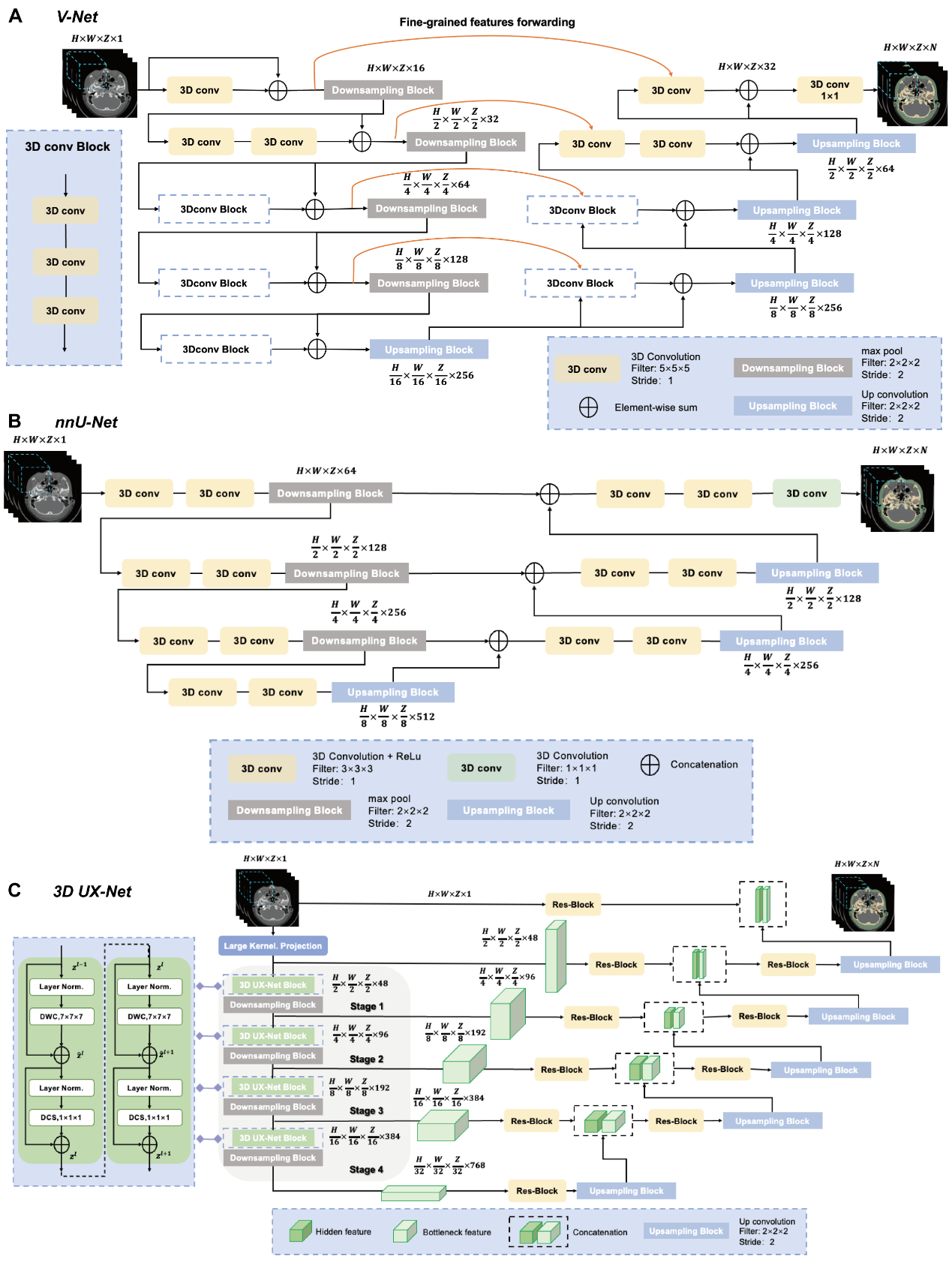


**Figure S4.** The framework of three backbones used in our study. (A) V-Net, (B) nnU-Net and (C) 3D UX-Net.

**Figure S5.** IoU for segmentation performance of CMF structures using V-Net, nnU-Net, and 3D UX-Net.


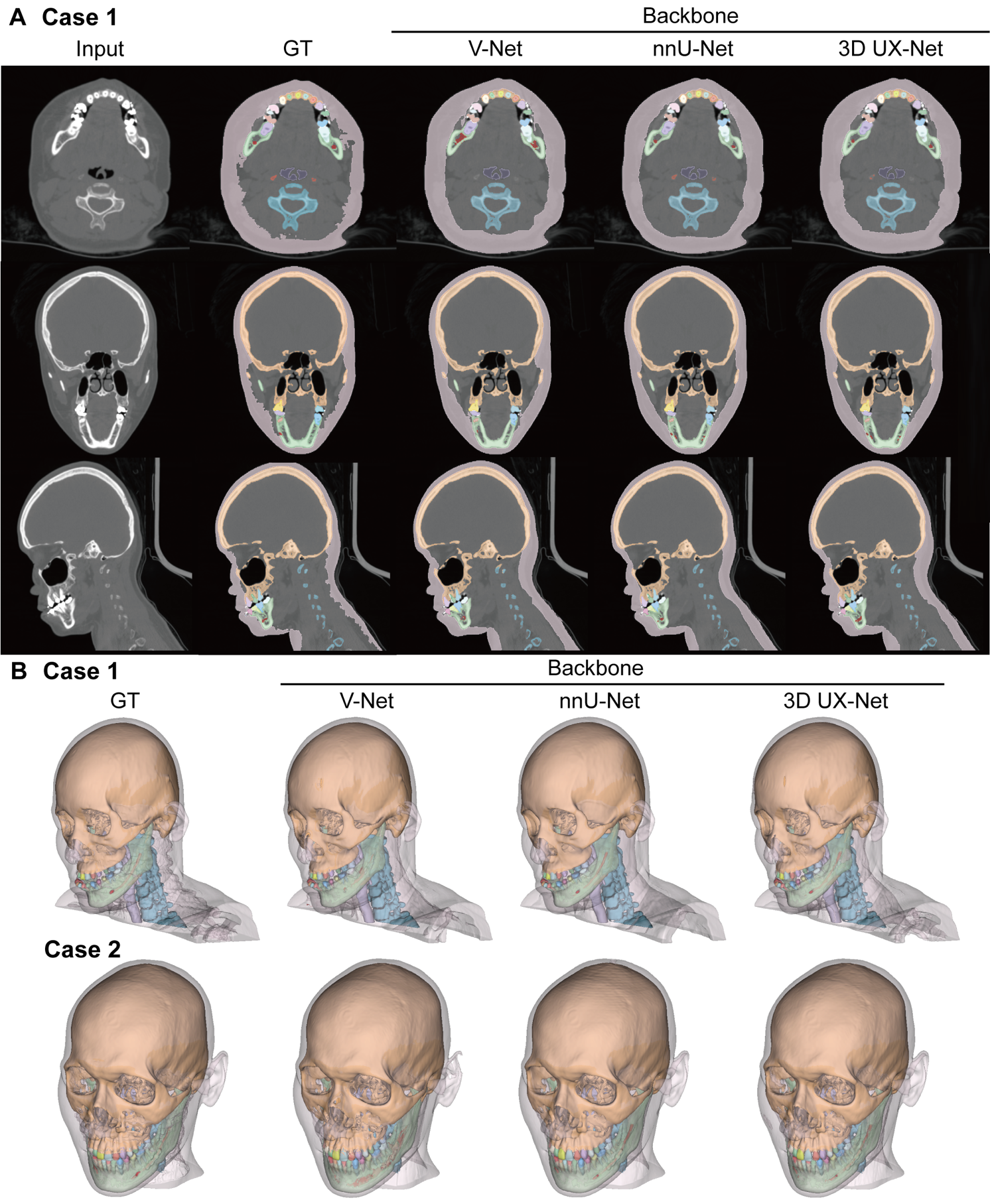


**Figure S6.** Segmentation results of CMF structures and individual teeth.

**
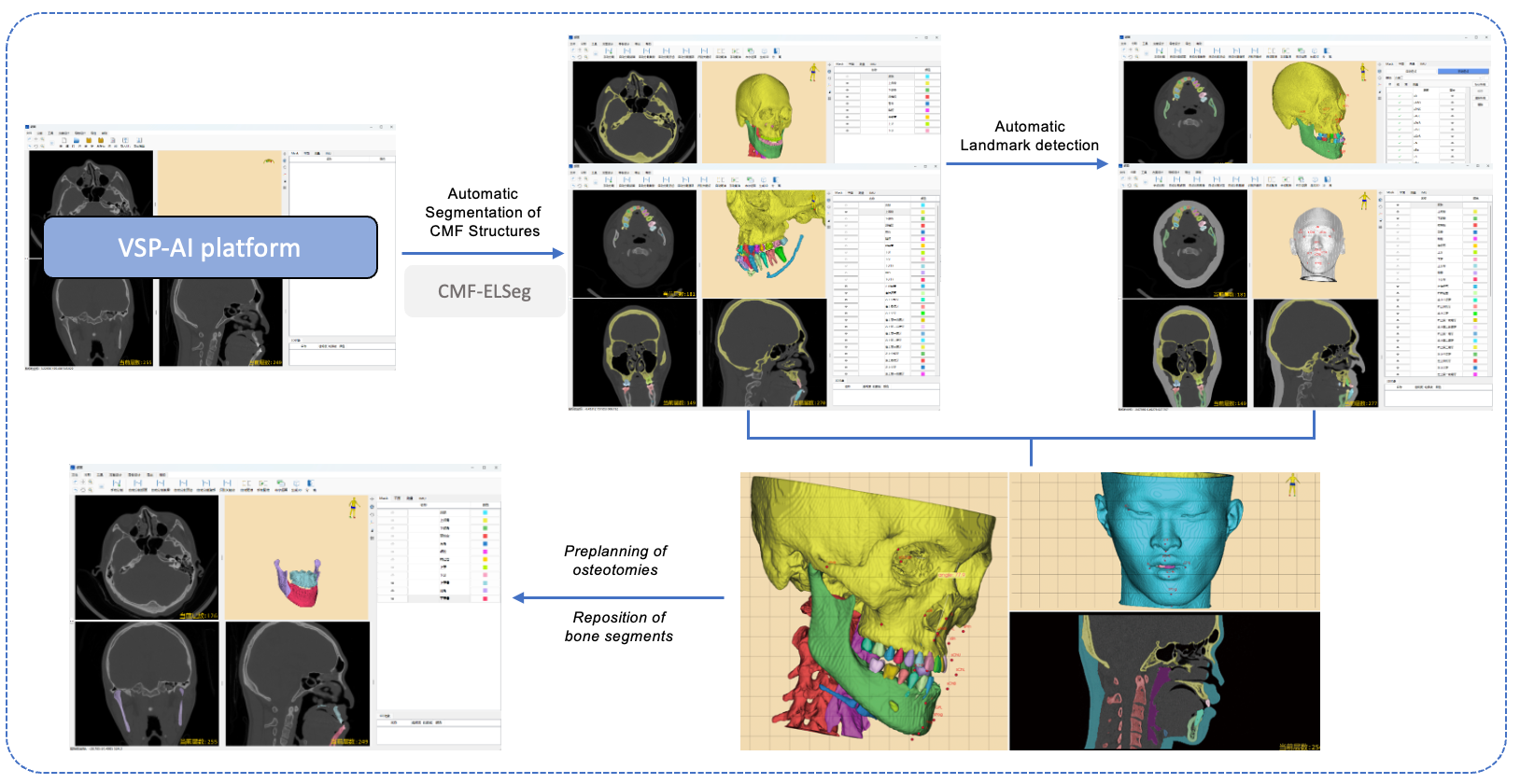
**

**Figure S7.** The workflow of VSP-AI platform and the application of CMF-ELSeg.

## Supplemental Tables

| **Table S1.** Sample characterization of Cohort 1. | |
| --- | --- |
| Characteristics | Overall |
| Gender, n (%) |  |
| Female | 53 (58.9%) |
| Male | 37 (41.1%) |
| Age, median (IQR) | 21 (20, 24) |
| Skeletal discrepancy, n (%) |  |
| Class II (4 ≤ ANB) | 12 (13.3%) |
| Class III (ANB < 0) | 52 (57.8%) |
| Class I (0 ≤ ANB < 4) | 26 (28.9%) |
| Facial asymmetry, n (%) |  |
| Without | 53 (58.9%) |
| With | 37 (41.1%) |
| Metal artifacts, n (%) |  |
| No | 43 (47.8%) |
| Yes | 47 (52.2%) |
| Orthodontic materials, n (%) |  |
| No | 50 (55.6%) |
| Yes | 40 (44.4%) |
| Metallic Crown, n (%) |  |
| No | 81 (90%) |
| Yes | 9 (10%) |
| Dental Implants, n (%) |  |
| No | 89 (98.9%) |
| Yes | 1 (1.1%) |
| Absence of maxillary anterior teeth, n (%) |  |
| No | 88 (97.8%) |
| Yes | 2 (2.2%) |
| Absence of mandibular anterior teeth, n (%) |  |
| No | 87 (96.7%) |
| Yes | 3 (3.3%) |
| Absence of maxillary premolar, n (%) |  |
| No | 55 (61.1%) |
| Yes | 35 (38.9%) |
| Absence of mandibular premolar, n (%) |  |
| No | 76 (84.4%) |
| Yes | 14 (15.6%) |
| Absence of maxillary 1st & 2nd molar, n (%) |  |
| No | 82 (91.1%) |
| Yes | 8 (8.9%) |
| Absence of mandibular 1st & 2nd molar, n (%) |  |
| No | 86 (95.6%) |
| Yes | 4 (4.4%) |
| Absence of maxillary 3rd molar, n (%) |  |
| No | 48 (53.3%) |
| Yes | 42 (46.7%) |
| Absence of mandibular 3rd molar, n (%) |  |
| No | 85 (94.4%) |
| Yes | 5 (5.6%) |

**Table S2.** Segmentation performance of V-Net, nnU-Net, and 3D UX-Net for CMF structures.

|  | | Dice | | IoU | |
| --- | --- | --- | --- | --- | --- |
|  |  | mean | Std | mean | Std |
| Facial soft tissue | V-Net | 0.9314 | 0.0415 | 0.8737 | 0.0572 |
|  | nnU-Net | 0.9329 | 0.0599 | 0.8783 | 0.0741 |
|  | 3DUX-Net | 0.9375 | 0.0470 | 0.8851 | 0.0628 |
| Upper skull | V-Net | 0.9740 | 0.0335 | 0.9509 | 0.0512 |
|  | nnU-Net | 0.9827 | 0.0143 | 0.9664 | 0.0257 |
|  | 3DUX-Net | 0.9838 | 0.0056 | 0.9682 | 0.0106 |
| Mandible bone | V-Net | 0.9452 | 0.0337 | 0.8978 | 0.0514 |
|  | nnU-Net | 0.9711 | 0.0099 | 0.9440 | 0.0183 |
|  | 3DUX-Net | 0.9704 | 0.0074 | 0.9425 | 0.0137 |
| Cervical vertebra | V-Net | 0.9604 | 0.0420 | 0.9264 | 0.0634 |
|  | nnU-Net | 0.9773 | 0.0152 | 0.9560 | 0.0280 |
|  | 3DUX-Net | 0.9779 | 0.0114 | 0.9569 | 0.0212 |
| Hyoid bone | V-Net | 0.7842 | 0.1107 | 0.6567 | 0.1331 |
|  | nnU-Net | 0.9251 | 0.0313 | 0.8622 | 0.0539 |
|  | 3DUX-Net | 0.9135 | 0.0801 | 0.8476 | 0.0941 |
| Pharyngeal cavity | V-Net | 0.9339 | 0.1040 | 0.8873 | 0.1188 |
|  | nnU-Net | 0.9665 | 0.0160 | 0.9356 | 0.0294 |
|  | 3DUX-Net | 0.9669 | 0.0146 | 0.9363 | 0.0270 |
| Inferior alveolar nerve | V-Net | 0.2932 | 0.0929 | 0.1752 | 0.0642 |
|  | nnU-Net | 0.7119 | 0.1257 | 0.5658 | 0.1362 |
|  | 3DUX-Net | 0.6179 | 0.1167 | 0.4566 | 0.1154 |
| Upper teeth | V-Net | 0.8766 | 0.0704 | 0.7855 | 0.0842 |
|  | nnU-Net | 0.9382 | 0.0206 | 0.8843 | 0.0351 |
|  | 3DUX-Net | 0.9256 | 0.0169 | 0.8619 | 0.0287 |
| Lower teeth | V-Net | 0.8547 | 0.0578 | 0.7499 | 0.0739 |
|  | nnU-Net | 0.9316 | 0.0284 | 0.8732 | 0.0467 |
|  | 3DUX-Net | 0.9171 | 0.0226 | 0.8477 | 0.0373 |

**Table S3.** Segmentation performance of cascaded segmentation networks based on V-Net, nnU-Net, and 3D UX-Net for individual teeth.

|  | | Dice | | IoU | |
| --- | --- | --- | --- | --- | --- |
|  |  | mean | Std | mean | Std |
| Maxillary central incisor | V-Net | 0.9419 | 0.0214 | 0.8909 | 0.0369 |
|  | nnU-Net | 0.9439 | 0.0226 | 0.8945 | 0.0386 |
|  | 3DUX-Net | 0.9471 | 0.0233 | 0.9007 | 0.0398 |
| Maxillary lateral incisor | V-Net | 0.9339 | 0.0245 | 0.8770 | 0.0410 |
|  | nnU-Net | 0.9377 | 0.0240 | 0.8836 | 0.0402 |
|  | 3DUX-Net | 0.9421 | 0.0264 | 0.8905 | 0.0436 |
| Maxillary cuspid | V-Net | 0.9417 | 0.0284 | 0.8911 | 0.0477 |
|  | nnU-Net | 0.9447 | 0.0284 | 0.8964 | 0.0476 |
|  | 3DUX-Net | 0.9469 | 0.0282 | 0.9015 | 0.0479 |
| Maxillary 1st premolar | V-Net | 0.9340 | 0.0280 | 0.8774 | 0.0464 |
|  | nnU-Net | 0.9402 | 0.0291 | 0.8885 | 0.0488 |
|  | 3DUX-Net | 0.9410 | 0.0284 | 0.8898 | 0.0483 |
| Maxillary 2nd premolar | V-Net | 0.9322 | 0.0464 | 0.8751 | 0.0685 |
|  | nnU-Net | 0.9401 | 0.0275 | 0.8882 | 0.0464 |
|  | 3DUX-Net | 0.9435 | 0.0324 | 0.8945 | 0.0527 |
| Maxillary 1st molar | V-Net | 0.9365 | 0.0381 | 0.8828 | 0.0587 |
|  | nnU-Net | 0.9430 | 0.0269 | 0.8932 | 0.0456 |
|  | 3DUX-Net | 0.9449 | 0.0314 | 0.8971 | 0.0516 |
| Maxillary 2nd molar | V-Net | 0.9360 | 0.0407 | 0.8824 | 0.0616 |
|  | nnU-Net | 0.9299 | 0.0534 | 0.8731 | 0.0822 |
|  | 3DUX-Net | 0.9440 | 0.0300 | 0.8956 | 0.0502 |
| Maxillary 3rd molar | V-Net | 0.9040 | 0.0874 | 0.8352 | 0.1156 |
|  | nnU-Net | 0.8749 | 0.1470 | 0.8000 | 0.1789 |
|  | 3DUX-Net | 0.9133 | 0.0777 | 0.8514 | 0.1153 |
| Mandibular central incisor | V-Net | 0.9093 | 0.0450 | 0.8365 | 0.0680 |
|  | nnU-Net | 0.9140 | 0.0478 | 0.8447 | 0.0714 |
|  | 3DUX-Net | 0.9171 | 0.0465 | 0.8495 | 0.0704 |
| Mandibular lateral incisor | V-Net | 0.9117 | 0.0576 | 0.8421 | 0.0816 |
|  | nnU-Net | 0.9150 | 0.0639 | 0.8480 | 0.0894 |
|  | 3DUX-Net | 0.9203 | 0.0577 | 0.8560 | 0.0814 |
| Mandibular cuspid | V-Net | 0.9346 | 0.0314 | 0.8787 | 0.0513 |
|  | nnU-Net | 0.9383 | 0.0302 | 0.8851 | 0.0499 |
|  | 3DUX-Net | 0.9390 | 0.0305 | 0.8872 | 0.0490 |
| Mandibular 1st premolar | V-Net | 0.9331 | 0.0337 | 0.8762 | 0.0549 |
|  | nnU-Net | 0.9344 | 0.0352 | 0.8792 | 0.0573 |
|  | 3DUX-Net | 0.9377 | 0.0285 | 0.8823 | 0.0504 |
| Mandibular 2nd premolar | V-Net | 0.9373 | 0.0207 | 0.8828 | 0.0353 |
|  | nnU-Net | 0.9405 | 0.0266 | 0.8884 | 0.0448 |
|  | 3DUX-Net | 0.9442 | 0.0233 | 0.8946 | 0.0393 |
| Mandibular 1st molar | V-Net | 0.9333 | 0.0423 | 0.8775 | 0.0649 |
|  | nnU-Net | 0.9407 | 0.0313 | 0.8892 | 0.0521 |
|  | 3DUX-Net | 0.9419 | 0.0305 | 0.8921 | 0.0509 |
| Mandibular 2nd molar | V-Net | 0.9285 | 0.0615 | 0.8718 | 0.0922 |
|  | nnU-Net | 0.9348 | 0.0505 | 0.8814 | 0.0786 |
|  | 3DUX-Net | 0.9421 | 0.0433 | 0.8970 | 0.0618 |

**Table S4.** Segmentation performance of CMF-ELSeg.

|  | Dice | | IoU | |
| --- | --- | --- | --- | --- |
|  | mean | Std | mean | Std |
| Facial soft tissue | 0.9420 | 0.0230 | 0.8912 | 0.0376 |
| Upper skull | 0.9879 | 0.0043 | 0.9761 | 0.0082 |
| Mandible bone | 0.9745 | 0.0071 | 0.9504 | 0.0132 |
| Cervical vertebra | 0.9827 | 0.0154 | 0.9665 | 0.0270 |
| Hyoid bone | 0.8320 | 0.1436 | 0.7346 | 0.1847 |
| Pharyngeal cavity | 0.9693 | 0.0133 | 0.9408 | 0.0248 |
| Inferior alveolar nerve | 0.6461 | 0.1069 | 0.4857 | 0.1096 |
| Maxillary central incisor | 0.9451 | 0.0247 | 0.8970 | 0.0424 |
| Maxillary lateral incisor | 0.9401 | 0.0268 | 0.8881 | 0.0452 |
| Maxillary cuspid | 0.9470 | 0.0282 | 0.9005 | 0.0480 |
| Maxillary 1st premolar | 0.9405 | 0.0282 | 0.8890 | 0.0480 |
| Maxillary 2nd premolar | 0.9422 | 0.0298 | 0.8914 | 0.0504 |
| Maxillary 1st molar | 0.9471 | 0.0255 | 0.9005 | 0.0439 |
| Maxillary 2nd molar | 0.9447 | 0.0282 | 0.8963 | 0.0481 |
| Maxillary 3rd molar | 0.9282 | 0.0515 | 0.8713 | 0.0801 |
| Mandibular central incisor | 0.9180 | 0.0425 | 0.8509 | 0.0660 |
| Mandibular lateral incisor | 0.9204 | 0.0577 | 0.8569 | 0.0825 |
| Mandibular cuspid | 0.9423 | 0.0307 | 0.8924 | 0.0512 |
| Mandibular 1st premolar | 0.9397 | 0.0332 | 0.8879 | 0.0548 |
| Mandibular 2nd premolar | 0.9437 | 0.0252 | 0.8962 | 0.0401 |
| Mandibular 1st molar | 0.9429 | 0.0361 | 0.8960 | 0.0480 |
| Mandibular 2nd molar | 0.9307 | 0.1053 | 0.8876 | 0.0814 |

| **Table S5.** Statistical analysis (*P* value) of segmentation performance between CMF-ELSeg and the baseline models. | | | | | | |
| --- | --- | --- | --- | --- | --- | --- |
|  | **Dice** | | | **IoU** | | |
| **Backbone of Cascaded segmentation networks** | **3D UX-Net** | **nnU-Net** | **V-Net** | **3D UX-Net** | **nnU-Net** | **V-Net** |
| Facial soft tissue | 0.3267 | 0.0416 | 6.19e-06 | 0.3267 | 0.0416 | 6.19e-06 |
| Upper skull | 1.01e-18 | 2.38e-09 | 3.96e-26 | 1.01e-18 | 2.38e-09 | 3.96e-26 |
| Mandible bone | 2.73e-08 | 0.0116 | 9.92e-28 | 2.73e-08 | 0.0116 | 9.92e-28 |
| Cervical vertebra | 2.51e-09 | 2.09e-06 | 3.74e-23 | 2.51e-09 | 2.09e-06 | 3.74e-23 |
| Hyoid bone | 8.57e-05 | 9.65e-06 | 7.12e-05 | 8.57e-05 | 9.65e-06 | 7.12e-05 |
| Pharyngeal cavity | 0.2615 | 0.3340 | 3.39e-12 | 0.2615 | 0.3340 | 3.39e-12 |
| Inferior alveolar nerve | 0.0677 | 2.96e-07 | 5.65e-29 | 0.0677 | 2.96e-07 | 5.65e-29 |
| Maxillary central incisor | 0.7707 | 0.1165 | 0.0070 | 0.6841 | 0.1165 | 0.0070 |
| Maxillary lateral incisor | 0.6098 | 0.0268 | 0.0005 | 0.8371 | 0.0268 | 0.0005 |
| Maxillary cuspid | 0.7125 | 0.0903 | 0.0038 | 0.9681 | 0.0903 | 0.0038 |
| Maxillary 1st premolar | 0.9631 | 0.7657 | 0.0015 | 0.9376 | 0.7657 | 0.0015 |
| Maxillary 2nd premolar | 0.8679 | 0.2489 | 0.0121 | 0.8102 | 0.3083 | 0.0125 |
| Maxillary 1st molar | 0.3325 | 0.0410 | 1.84e-05 | 0.3536 | 0.0385 | 2.47e-05 |
| Maxillary 2nd molar | 0.6067 | 0.0093 | 0.0013 | 0.6625 | 0.0093 | 0.0016 |
| Maxillary 3rd molar | 0.1811 | 0.0740 | 0.0027 | 0.3386 | 0.0536 | 0.0017 |
| Mandibular central incisor | 0.9821 | 0.4252 | 0.0021 | 0.8603 | 0.4304 | 0.0021 |
| Mandibular lateral incisor | 0.8836 | 0.0984 | 0.0006 | 0.6349 | 0.0870 | 0.0006 |
| Mandibular cuspid | 0.0776 | 0.0169 | 0.0001 | 0.0920 | 0.0162 | 0.0001 |
| Mandibular 1st premolar | 0.0605 | 0.0254 | 0.0008 | 0.0561 | 0.0276 | 0.0008 |
| Mandibular 2nd premolar | 0.5730 | 0.0721 | 0.0002 | 0.3644 | 0.0489 | 9.1e-05 |
| Mandibular 1st molar | 0.4633 | 0.1754 | 0.0003 | 0.4558 | 0.1594 | 0.0003 |
| Mandibular 2nd molar | 0.9340 | 0.0654 | 0.0082 | 0.8649 | 0.0597 | 0.0081 |

**Table S6.** Qualitative results and revision times of automatic segmentation.

|  | Dice | | Revision time (min) | |
| --- | --- | --- | --- | --- |
|  | mean | Std | mean | Std |
| Facial soft tissue | 0.99731284 | 0.01448083 | 2.35714286 | 1.31384568 |
| Upper skull | 0.99874931 | 0.00423691 | 3.8 | 1.64316767 |
| Mandible bone | 0.99973679 | 0.00144166 | 2* | / |
| Hyoid bone | 0.94327382 | 0.14846359 | 4.25 | 2.85919569 |
| Pharyngeal cavity | 0.99780018 | 0.00838072 | 6 | 1.41421356 |
| Inferior alveolar nerve | 0.88169256 | 0.15321339 | 6.27777778 | 3.61053288 |
| Upper teeth | 0.98384078 | 0.02676791 | 5.625 | 3.3106234 |
| Lower teeth | 0.97664002 | 0.036888 | 4.42857143 | 3.22762799 |

- *The count was a single case; no standard deviation was calculated.
